# Glucagon-like peptide 1 improves insulin resistance *in vitro* through anti-inflammation of macrophages

**DOI:** 10.1590/1414-431X20165826

**Published:** 2016-11-21

**Authors:** C. Guo, T. Huang, A. Chen, X. Chen, L. Wang, F. Shen, X. Gu

**Affiliations:** Department of Endocrinology, The First Affiliated Hospital of Wenzhou Medical University, Wenzhou, Zhejiang, China

**Keywords:** Diabetes, Glucagon-like peptide, Macrophage infiltration, Adipose inflammation, Insulin resistance

## Abstract

Glucagon-like peptide 1 (GLP-1), a kind of gut hormone, is used in the treatment of type 2 diabetes (T2D). Emerging evidence indicates that GLP-1 has anti-inflammatory activity. Chronic inflammation in the adipose tissue of obese individuals is a cause of insulin resistance and T2D. We hypothesized that GLP-1 analogue therapy in patients with T2D could suppress the inflammatory response of macrophages, and therefore inhibit insulin resistance. Our results showed that GLP-1 agonist (exendin-4) not only attenuated macrophage infiltration, but also inhibited the macrophage secretion of inflammatory cytokines including TNF-β, IL-6, and IL-1β. Furthermore, we observed that lipopolysaccharide (LPS)-induced macrophage conditioned media could impair insulin-stimulated glucose uptake. This effect was compensated by treatment with the conditioned media from macrophages treated with the combination of LPS and exendin-4. It was also observed that exendin-4 directly inhibited the activation of NF-κB in macrophages. In conclusion, our results indicated that GLP-1 improved inflammatory macrophage-derived insulin resistance by inhibiting NF-κB pathway and secretion of inflammatory cytokines in macrophages. Furthermore, our observations suggested that the anti-inflammatory effect of GLP-1 on macrophages can contribute to GLP-1 analogue therapy of T2D.

## Introduction

Obesity and type 2 diabetes (T2D) are considered chronic pro-inflammatory diseases (1). Adipose tissue macrophages (ATMs) produce inflammatory cytokines and play an important role in such chronic inflammatory responses (2,3). Macrophages that are mostly polarized have either an M1 or an M2 phenotype (4). In general, the activation status of M1 or M2 phenotype are influenced by local microenvironments (5,6). Th1 cytokines (such as IFN-γ) induce activation of M1 macrophages, which produce inflammatory mediators. Th2 cytokines (such as IL-4 and IL-13) induce the alternative activation of M2 macrophages, which regulate anti-inflammatory responses. Accumulated evidence has proven that macrophage polarization plays a critical role in the development of T2D. It is considered that the imbalance in the M1/M2 macrophage ratio leads to chronic inflammation in adipose tissue of T2D patients (1). The majority of ATMs in lean individuals exhibit an anti-inflammatory M2 polarity. Accumulation of M1 macrophages in obesity individuals are associated with insulin resistance (1,7,8).

Obesity-related insulin resistance plays a critical role in the cause and development of diabetic pathophysiology (9). The infiltration of macrophages into adipose tissue is the initial event of inflammation in the adipose tissues (10). M2 macrophages produce anti-inflammatory cytokines, such as IL-10. It is reported that M2 macrophages can increase insulin-dependent glucose uptake in adipocytes of lean people (11). M1 macrophages, which represent a pro-inflammatory state, can secrete inflammatory cytokines such as tumor necrosis factor (TNF)-α, interleukin-6 (IL-6) and IL-1β that interfere with insulin signaling, and cause adipocyte dysfunction and insulin resistance (4,10).

NF-κB pathway plays a key role in inflammation by its ability to induce transcription of pro-inflammatory genes (12). The NF-κB family is formed by several members, including NF-κB1 (p50/p105), NF-κB2 (p52/p100), p65 (RelA), RelB, and c-Rel (13). The activity of NF-κB is regulated by inhibitors of κB (IκB) proteins and IκB kinase (IKK). NF-κB is located in the cytoplasm in an inactive form, which associates with IκB. The most important form of IκB is IκBα, IκBβ, and IκBε. Phosphorylation of IκB that is mediated by IKK is an important step in NF-κB, which causes dissociation of NF-κB and IκB and its translocation into nuclei (14). As one of the most important regulators of pro-inflammatory gene expression, NF-κB regulates the synthesis of many inflammatory cytokines including TNF-α, IL-1β, IL-6 and IL-8 (14,15).

Glucagon-like peptide-1 (GLP-1), a kind of incretin hormone, is secreted by intestinal L cells in response to nutrients. Then, GLP-1 stimulates the release of insulin from pancreatic β-cells (16). It has been reported that the secretion of GLP-1 decreases in patients with T2D (17). Now, the GLP-1 analogues have been used in combination with insulin for patients with T2D. It has also been reported that a GLP-1 agonist plays an anti-inflammatory function in cultured human macrophages (18). Moreover, GLP can inhibit adipose tissue macrophage infiltration and inflammation in an obese mouse model of diabetes (19). However, whether GLP-1 affects insulin resistance by suppressing macrophage inflammation is not clear.

In the present study, we evaluated the effects of exendin-4, a kind of GLP-1 analogue, in the infiltration of macrophages, and in the secretion of inflammatory cytokines including TNF-α, IL-6, and IL-1β by inactivation of NF-κB pathway. The inhibitory effect of GLP-1 on macrophage inflammation further prevents the impairment of insulin sensitivity induced by lipopolysaccharide (LPS)-stimulated inflammation. Our results that GLP-1 improved insulin resistance by anti-inflammation of macrophages provide a new biological mechanism for the clinical therapeutics of T2D.

## Material and Methods

### Cell culture and cell transfection

Mouse peritoneal macrophages were prepared, isolated and cultured as described previously (20). The mouse macrophage cell line (RAW264) and mouse preadipocyte (3T3-L1s) were obtained from Shanghai Institute of Chinese Academy of Sciences (China). RAW264 cells were maintained in DMEM (Gibco, USA), supplemented with 10% fetal bovine serum at 37°C in a humidity incubator within 5% CO_2_. 3T3-L1 cells were cultured in DMEM, supplemented with 10% calf serum at 37°C in a humidity incubator within 5% CO_2_. RAW264 cells were grown in 6-well plates with a 75% confluence at 24 h before transfection. Cell transfection was carried out using lipofectamine2000 (Invitrogen, USA) according to the manufacturer’s description. RAW264 cells and mouse peritoneal macrophages were transfected with siRNA of GLP-1 receptor (GLP-1R) and scramble siRNA. The sequences of siRNA were described in previous studies (21,22): GLP-1R forward, 5′-AUA AUG AGC CAG UAG UUC AUG UUGG-3′ and reverse, 5′-CCA ACA UGA ACU ACU GGC UCA UUAU-3′; negative control (scramble) forward, 5′-UUC UCC GAA CGU GUC ACG UTT-3′; reverse, 5′-ACG UGA CAC GUU CGG AGA ATT-3′. These sequences were synthesized by GenePharma Co. (China).

### Protein extraction and western blot

Protein extraction and western blot were performed as previously described (23). Briefly, total proteins were extracted from RAW264 cells and mouse peritoneal macrophages using a RIPA lysis buffer (Beyotime Biotech Inc., China) supplemented with Complete EDTA-free protease inhibitor cocktail tablets (Roche, USA) according to the manufacturer’s instructions. Total protein concentrations were assayed with the BCA protein assay kit (Applygen, China). The protein concentration was diluted to 2 μg/μL in every sample. Approximately 40 μg of protein in every sample were fractionated by gel electrophoresis, and transferred to a polyvinylidene fluoride membrane (Millipore, USA), blocked with 5% skim milk for 1 h, and then incubated with primary antibodies including anti-IκBα, anti-p-IκBα, anti-NF-κB, anti-GLP-1R, and anti-β-actin (Santa Cruz, USA) at 4°C overnight. The membrane was next incubated with horseradish peroxidase-conjugated secondary antibodies for 1 h after three washes with TBST. Signals were tested by enhanced chemiluminescence detection reagent (Thermo Scientific, USA) and protein expressions were calculated by normalization to β-actin. All detection reactions were repeated three times. Western blots showed in the figures are representative of three independent experiments.

### RNA extraction and qPCR

Total RNA was isolated from RAW264 cells using Trizol (Invitrogen) under the manufacturer’s instructions and then cDNA was generated from RNA using a First Strand cDNA Synthesis Kit (Fermentas, USA). Quantitative real-time PCR (qRT-PCR) was performed using 2×TransStart Green qPCR SuperMix (TransGen Biotech Co., China) on an ABI 7300 instrument as previously described (24). mRNA levels were normalized to the level of β-actin. PCRs were performed in duplicates, and error bars in the charts represent the corresponding standard deviations. The primers used to detect mouse GLP-1R (22) were forward: 5′-TTG GGG TGA ACT TCC TCA TC-3′, reverse: 5′-CTT GGC AAG TCT GCA TTT GA-3′; β-actin (25) forward: 5′-GCC AAC CGT GAA AAG ATG ACC-3′, and reverse: 5′-CCC TCG TAG ATG GGC ACA GT-3′.

### Transwell assays

Transwell migration assay of macrophage was performed as previously described (25). Briefly, 2×10^5^ RAW264 cells or mouse peritoneal macrophages transfected with scramble or GLP-1R siRNAs were supplemented with 100 μL serum-free medium and placed in the upper chamber with 8-mm pore size (BD Bioscience, USA) for migration assays, while 600 μL serum-free medium with or without LPS (200 ng/mL) were placed in the lower chamber. One of LPS groups was added with exendin-4 into the upper chamber. After incubation for 24 h at 37°C, the cells in the upper membrane were discarded and cells on the lower membrane were fixed using 95% ethanol and stained with crystal violet (Beyotime). Next, five random fields were counted. Each experiment was performed in triplicate. Migrated cell number was measured with Image-Pro Plus 6.0 software (Media Cybernetics, USA).

### Macrophage-conditioned media (CM) preparation and inflammatory factors detection

Seventy to eighty percent confluence of RAW264 cells or mouse peritoneal macrophages in 6-well plates were starved with serum-free medium, or serum-free DMEM containing LPS (200 ng/mL) with or without exendin-4 (2.5 nM; Sigma, USA) overnight. Then the cells were washed for three times using PBS, and incubated in serum-free medium for 24 h. The cells cultured in conditioned media were collected, centrifuged at 500 *g* for 5 min at 4^o^C, filtered through a 0.22-μm syringe filter, and stored at 4°C before being used for the experiments. RAW264 cells or mouse peritoneal macrophages starved with serum-free medium were defined as macrophage-conditioned media (CM). CM from macrophages treated with serum-free DMEM containing LPS with or without exendin-4 (2.5 nM; Sigma) were defined as CM-LPS-Ex4 or CM-LPS, respectively.

The levels of TNF-α, IL-6, and IL-1β in conditioned media were investigated using enzyme-linked immunosorbent assay (ELISA). The concentration of these factors was measured using a Human Quantikine ELISA kit (R&D Systems, USA), according to the manufacturer’s instructions.

### Insulin-stimulated glucose uptake

3T3-L1 adipocytes were used for determining insulin-stimulated glucose uptake as previously described (26). Briefly, the 3T3-L1 preadipocytes were differentiated into adipocytes as described in a previous report (25). After differentiation, the medium was switched to low-glucose DMEM containing 0.3% bovine serum albumin (BSA) alone (control group) or with CM, CM-LPS, or CM-LPS-Ex4 and incubated at 37°C for 16 h. Then, the medium was switched to a KRBH buffer containing 10 nM of insulin with or without vehicles (DMSO), and with methanol and water extracts, and further incubated at 37°C for 30 min. After incubation, 0.1 lCi 2-deoxy-D-[^3^H] glucose was added into the KRBH buffer for 10 min. At the end of the incubation, the buffer was removed and the cells were washed three times with ice-cold PBS. The radioactivity of ^3^H was counted using a Wallac Liquid Scintillation Counter (USA) to determine glucose uptake. Non-specific glucose uptake was measured in cells treated with vehicles and with methanol and water extracts without insulin.

### Statistical analyses

Data are reported as means±SD. Student’s *t*-test was performed to assess differences between two groups. One-way ANOVA was conducted to assess differences among multiple groups. All statistical calculations were carried out using SPSS 19.0 software (USA) and P<0.05 was considered statistically significant.

## Results

### GLP-1R knockdown with siRNA in macrophages

**Figure 1:**
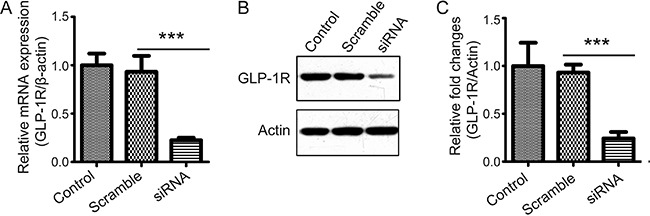
GLP-1R knockdown in macrophages using siRNA. RAW264 cells were transfected with siRNA of GLP-1 receptor (GLP-1R) and scramble siRNA for 72 h. *A*, The mRNA levels of GLP-1R normalized to β-actin were assessed by qPCR. Data are reported as relative values compared to the control group (n=6). *B*, Protein levels of GLP-1R assessed by western blot. *C*, Relative fold changes by western blot. The results are representative of three independent experiments. Data are reported as means±SD. ***P<0.001 (ANOVA).

To evaluate the function of GLP-1, siRNA was used to knockdown GLP-1R in macrophages. Then, the knockdown effect of GLP-1R siRNA was detected by qPCR and western blot. The results showed that the expression levels of GLP-1R were significantly decreased (Figure 1A-C). Therefore, siRNA for GLP-1R can be used to analyze the function of GLP in macrophages.

### Exendin-4 inhibited the migration of macrophages

**Figure 2:**
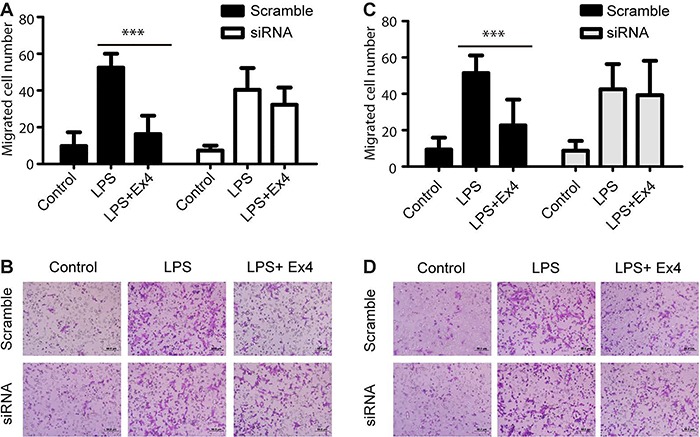
Effect of exendin-4 (Ex4) on the migration of macrophages and corresponding photomicrographs (bar: 50 μm). RAW264 cells (*A* and *B*) or mouse peritoneal macrophages (*C* and *D*) were treated with lipopolysaccharide (LPS) or a combination of LPS and exendin-4, and then transwell migration was performed for 24 h. Data are reported as means±SD. ***P<0.001 (ANOVA).

The infiltration of macrophages initiates low-grade inflammation in the adipose tissues, which is an important factor in the development of diabetes (27). To determine whether GLP affects macrophage infiltration, we treated RAW264 cells or mouse peritoneal macrophages with LPS, and tested whether LPS treatment-induced macrophages migration was inhibited by exendin-4 that is a long-acting potent agonist of GLP-1R (28). Our results showed that LPS led to an increased transwell migration of RAW264 cells and mouse peritoneal macrophages, and this effect was reversed by exendin-4 treatment (Figure 2A-D). Moreover, knocking down of GLP-1R using siRNA could rescue the inhibitory effect of exendin-4 on macrophage migration (Figure 2A-D). These results suggest that GLP can suppress LPS-induced macrophage infiltration.

### Exendin-4 inhibited the secretion of inflammatory factors

**Figure 3:**
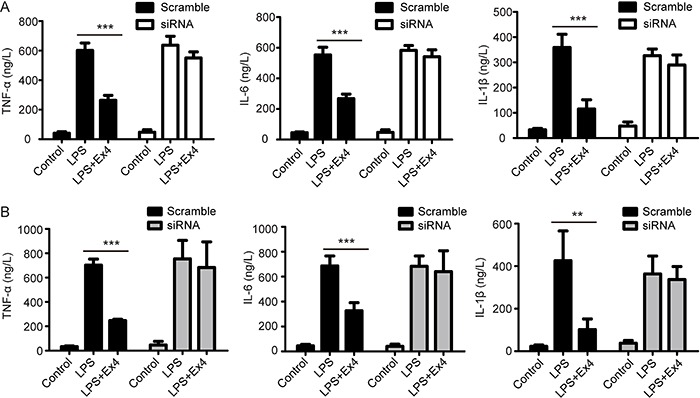
Effect of exendin-4 (Ex4) on the secretion of inflammatory macrophage cytokines. RAW264 cells (*A*) or mouse peritoneal macrophages (*B*) were treated with lipopolysaccharide (LPS) or a combination of LPS and Ex4 overnight, and then incubated in serum-free medium for 24 h. The secretion of TNF-α, IL-6, and IL-1β was evaluated using enzyme-linked immunosorbent assay (ELISA). Data are reported as means±SD. **P<0.01; ***P<0.001 (ANOVA).

Inflammatory cytokines such as TNF-α, IL-6, and IL-1β can cause adipocyte dysfunction, which is involved in the development of diabetes. To study the effect of GLP-1 on the macrophage-secreted inflammatory cytokines, we detected the levels of TNF-α, IL-6, and IL-1β in RAW264 cells and mouse peritoneal macrophages after treatment with exendin-4. As showed in Figure 3A and B, LPS treatment induced the upregulation of TNF-α, IL-6, and IL-1β in RAW264 and mouse peritoneal macrophages, which could be rescued by exendin-4 treatment. In addition, knocking down GLP-1R in RAW264 cells and mouse peritoneal macrophages using siRNA could reverse the inhibitory effect of exendin-4 on secretion of TNF-α, IL-6, and IL-1β (Figure 3A and B). Taken together, all of these results suggest that GLP could reduce LPS-induced inflammatory cytokines secretion of macrophages.

### Exendin-4 increased insulin-stimulated glucose uptake by targeting inflammatory macrophages

**Figure 4:**
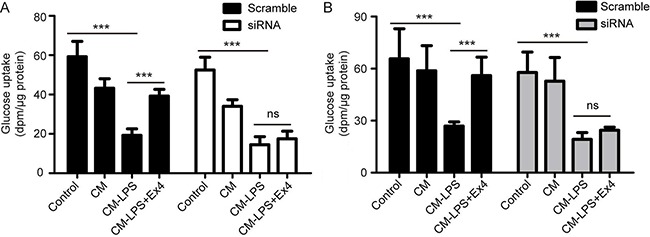
Effect of exendin-4 (Ex4)-treated macrophage conditioned media (CM) on insulin-stimulated glucose uptake. 3T3-L1 adipocytes were untreated (control group) or treated with CM, CM-lipopolysaccharide (LPS), or CM-LPS-Ex4 from RAW264 cells (*A*) or mouse peritoneal macrophages (*B*). The results are representative of three independent experiments. Data are reported as means±SD. ***P<0.001 (ANOVA). ns: non-significant.

It was reported that adipose tissue inflammation in the prediabetic state is related to increased insulin resistance (29,30). To explore the effect of exendin-4 on macrophage-secreted inflammatory cytokines mediated insulin resistance, we tested the insulin-stimulated glucose uptake using 3T3-L1 adipocytes which were incubated with LPS or exendin-4-treated macrophage CM from RAW264 cells or mouse peritoneal macrophages. Our results showed that macrophage CM and LPS-treated macrophage CM reduced insulin-stimulated glucose uptake (Figure 4A and B). Moreover, CM from exendin-4 and LPS-treated macrophages could rescue the inhibitory effect of CM-LPS on insulin-stimulated glucose uptake, which could be abrogated by GLP-1R knockdown in macrophages (Figure 4A and B). Taken together, these results suggest that GLP inhibited macrophage-secreted inflammatory factors and induced insulin resistance *in vitro*.

### Exendin-4 inhibited activation of NF-κB in macrophages

**Figure 5:**
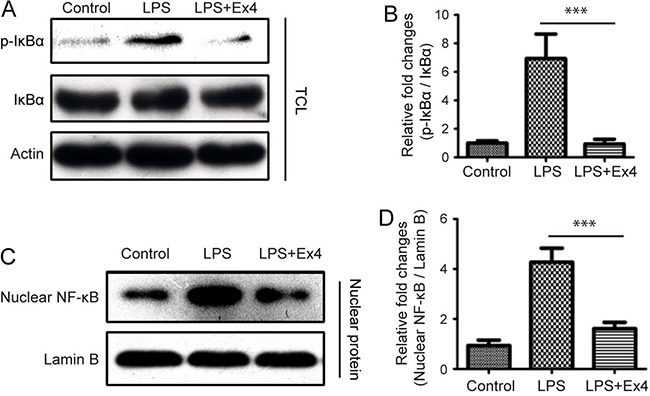
Effect of exendin-4 (Ex4) on lipopolysaccharide (LPS)-activated NF-κB pathway in RAW264 macrophages. RAW264 cells were treated with LPS or a combination of LPS and Ex4 for 30 min. Western blot image (*A*) and quantification (*B*) of basal protein and phosphorylation levels of IκBα. Western blot image (*C)* and quantification (*D*) of NF-κB protein level in nuclei. TCL: total cell lysate. The results are representative of three independent experiments. Data are reported as means±SD. ***P<0.001 (ANOVA).

**Figure 6:**
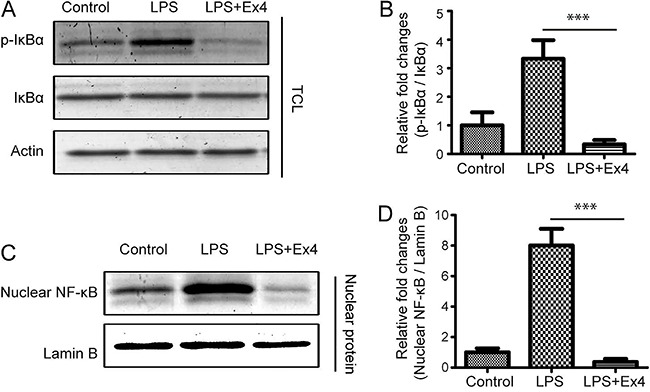
Effect of exendin-4 (Ex4) on lipopolysaccharide (LPS)-activated NF-κB pathway in mouse peritoneal macrophage. Mouse peritoneal macrophages were treated with LPS or a combination of LPS and Ex4 for 30 min. Western blot image (*A*) and quantification (*B*) of basal protein and phosphorylation levels of IκBα. Western blot image (*C)* and quantification (*D*) of NF-κB protein level in nuclei. TCL: total cell lysate. The results are representative of three independent experiments. Data are reported as means±SD. ***P<0.001 (ANOVA).

All of the above results suggested that GLP inhibited insulin resistance by preventing the inflammation response of macrophages. It was reported that activation of NF-κB is involved in the secretion of inflammatory cytokines. To investigate the mechanism of GLP-suppressed inflammation, we examine the expression and phosphorylation level of IκBα, and the nuclear translocation of NF-κB in RAW246 cells and mouse peritoneal macrophages. Our results showed that LPS did not affect the expression levels of IκBα, but increased the phosphorylation of IκBα and the nuclear translocation of NF-κB (Figures 5 and 6), which suggests that LPS could activate the transcription factor NF-κB. Furthermore, we found that exendin-4 treatment could reverse the activation of NF-κB induced by LPS (Figures 5 and 6). Thus, we infer that GLP likely inhibits the secretion of inflammatory cytokines by inactivation of NF-κB pathway.

## Discussion

T2D represents a significant threat to global health and human life (31). Insulin resistance is an important contributor of T2D. Chronic inflammation is a major cause of insulin resistance (27,32). The infiltration and accumulation of macrophages drive inflammation in the adipose tissues, and induced the secretion of inflammatory cytokines such as TNFα, which in turn lead to adipocyte dysfunction (33). Thus, inhibition of macrophage infiltration and adipose inflammation is an important target for clinical treatment of insulin resistance and T2D.

GLP-1 is a gut hormone, which can increase pancreatic secretion of insulin and be used in the clinical therapy of T2D (16). In the current study, we found that exendin-4 showed an inhibitory effect on migration of RAW264 macrophages. This result suggests that GLP could directly target macrophages and suppress its infiltration. Consistently, previous studies have reported that GLP-1 inhibits macrophage infiltration in adipose tissue (19), liver and vessel wall (34).

Chronic inflammation plays a critical role in the development of insulin resistance. Proinflammatory cytokines are mainly secreted by inflammatory macrophages in adipose tissue (16). Our results showed that GLP-1 treatment reduced the release of the inflammatory cytokines TNF-α, IL-6, and IL-1β in LPS-stimulated macrophages. These results are consistent with previous observations that GLP-1 inhibits the secretion of IL-1β and TNF-α *in vitro* (18) as well as IL-1β and IL-6 *in vivo* (35). Our observations taken together with previous studies suggest that GLP-1 has anti-inflammatory properties in macrophages. It has been indicated that proinflammatory cytokines can directly affect insulin signaling pathway and impair insulin sensitivity (36). Bouzakri and Zierath (37) reported that TNF-α leads to insulin resistance by directly targeting muscle insulin signaling. Accordingly, our results showed that LPS-treated macrophage CM decreased the insulin-stimulated glucose uptake in 3T3-L1 adipocytes, and this effect was reversed by CM from macrophages treated with exendin-4. These results suggest that GLP-1 increases insulin sensitivity by inhibiting the production of inflammatory cytokines in macrophages.

It has been well documented that activation of NF-κB plays a central role in inflammatory events (12,14). Our results showed that GLP-1 inhibited the activation of NF-κB pathway in macrophages, which is consistent with a previous *in vivo* study (19). Another study demonstrated that the main effects of GLP-1 are regulated by the activation of adenylate cyclase and the production of cAMP (38). Meanwhile, cAMP/PKA pathway regulates inflammatory response of macrophages via inhibiting the production of proinflammatory cytokines (39,40). Arakawa et al. have indicated that LPS-induced macrophage activation and TNF-α expression was significantly reduced by GLP-1 analog exendin-4 through PKA/NF-κB signaling pathway (20). Moreover, It has been reported that activation of NF-κB can stimulate the transcription of proinflammatory genes including TNF-α, IL-1β, IL-6, and IL-8 (14). Taken together, our results and previous observations suggest that GLP-1 inhibits inflammatory response of macrophages and the production of TNF-α, IL-6, and IL-1β by cAMP/PKA/NF-κB signaling pathway.

Although the improving effect of GLP-1 on insulin resistance by targeting inflammatory macrophages was demonstrated in this study, only RAW246 macrophage cell line, mouse peritoneal macrophages and 3T3-L1 adipocytes were used *in vitro*. Therefore, there are some limitations in our study. In future researches, whether GLP-1 inhibits inflammatory response of macrophages and therefore improves insulin resistance *in vivo* needs to be further investigated.

In conclusion, our findings suggest that GLP-1 analogue had inhibitory effects on macrophage-mediated adipose tissue inflammation and could be used for therapy in patients with T2D.
